# Incidence of self-reported brain injury and the relationship with substance abuse: findings from a longitudinal community survey

**DOI:** 10.1186/1471-2458-10-171

**Published:** 2010-03-29

**Authors:** Robert J Tait, Kaarin J Anstey, Peter Butterworth

**Affiliations:** 1 Centre for Mental Health Research, Australian National University. Canberra, ACT, 0200 Australia

## Abstract

**Background:**

Traumatic or serious brain injury (BI) has persistent and well documented adverse outcomes, yet 'mild' or 'moderate' BI, which often does not result in hospital treatment, accounts for half the total days of disability attributed to BI. There are currently few data available from community samples on the incidence and correlates of these injuries. Therefore, the study aimed to assess the 1) incidence of self-reported mild (not requiring hospital admission) and moderate (admitted to hospital)) brain injury (BI), 2) causes of injury 3) physical health scores and 4) relationship between BI and problematic alcohol or marijuana use.

**Methods:**

An Australian community sequential-cohort study (cohorts aged 20-24, 40-44 and 60-64 years at wave one) used a survey methodology to assess BI and substance use at baseline and four years later.

**Results:**

Of the 7485 wave one participants, 89.7% were re-interviewed at wave two. There were 56 mild (230.8/100000 person-years) and 44 moderate BI (180.5/100000 person-years) reported between waves one and two. Males and those in the 20-24 year cohort had increased risk of BI. Sports injury was the most frequent cause of BI (40/100) with traffic accidents being a greater proportion of moderate (27%) than mild (7%) BI. Neither alcohol nor marijuana problems at wave one were predictors of BI. BI was not a predictor of developing substance use problems by wave two.

**Conclusions:**

BI were prevalent in this community sample, though the incidence declined with age. Factors associated with BI in community samples differ from those reported in clinical samples (e.g. typically traumatic brain injury with traffic accidents the predominate cause). Further, detailed evaluation of the health consequences of these injuries is warranted.

## Background

Traumatic brain injury (TBI - in this paper used to differentiate the most serious cases) is a major cause of morbidity and mortality in developed countries, with recent estimates from the USA that there were about 90 TBI-related hospitalizations per 100000 people [1,2] although others cite higher typical values (e.g. 200-300 per 100000 [3]). The importance of TBI is the persistence extent of the associated disability. TBI impacts on a wide range of domains including cognitive function [4], depression [5] and suicidality [6] with 80% of TBI cases likely to have one Axis I disorder and nearly half of TBI cases likely to have two or more mental health disorders [7]. TBI can also result in major changes in personality and behaviour with deleterious consequences for relationships, employment and social circumstances [8].

In addition to head trauma resulting in hospitalization, there is an increasing realization that many of those with BI are treated in emergency departments or primary care, possibly after a long delay (weeks to months) or do not receive any medical attention [9,10]. In the USA, a national survey estimated that 381 000 people a year did not seek medical care after a brain injury, 221 000 were treated in primary care and 543 000 in emergency departments [11]. Taking this into account, the overall TBI incident rate was estimated at 618 per 100000 [11]. While these (non-hospitalised) injuries are sometimes referred to as 'mild' or 'moderate', their consequences can still be substantial; for example, they account for half the days of disability each year attributed to brain injury [12].

The relationship between alcohol use and brain injuries has been well documented. Over one-third of patients hospitalized with brain injuries are likely to be intoxicated at admission and 44% to 66% have a history of alcohol abuse [13]. Over a third of those receiving treatment for TBI also report or test positive for illicit drugs use [14]. Post-injury substance use is less clear cut. A decline in alcohol related problems has been reported in the 12-months after injury, but the extent of this decline is difficult to separate from the base rate of change, that is, those with alcohol disorders in the wider population also cycle through periods of reduced alcohol problems [15]. Nevertheless, pre-injury alcohol problems are predictive of post-injury problems, with few people who were abstainers or light drinkers, subsequently developing alcohol use problems [15].

One reason why knowledge on the relationship between substance use and brain injuries may not be representative of the whole population is that data are typically drawn from hospital or rehabilitation settings [13-16] where the prevalence of alcohol intoxication or those with alcohol disorders is likely to be over-represented. Among those hospitalised with TBI, motor vehicle accidents are likely to be the primary source of injury [3,17,18]. For those experiencing a motor vehicle accident, alcohol use is associated with more severe injuries and a greater proportion of crashes classified as severe occur in those who are intoxicated [19]. By comparison, in ambulatory settings the prevalence of falls, assaults and 'other' causes predominate [10,11,20].

There are few data from community surveys examining BI or TBI, particularly from outside the United States of America and a lack of longitudinal follow-up. In 2004, a cross-sectional cohort study reported that the life-time prevalence of brain injuries resulting in at least 15 minutes loss of consciousness was approximately 6% with a five year prevalence of 2.5% in those aged 20-24 years, 0.4% in the 40-44 year cohort and 0.2% in the oldest cohort, aged 60-64 years [21]. The aim of the current study was to assess the incidence of first-time self-reported brain injuries over the subsequent four years for each of these three cohorts, causes of these injuries and the associated change in physical health scores. In addition, this study also sought to examine the relationship between alcohol or marijuana problems at wave one and subsequent brain injuries. Finally, the development of substance use problems at wave two for those who incurred brain injuries was evaluated.

## Method

### Participants

The sample for the PATH Through Life Project has previously been described in detail [21,22] and has been shown to be representative of the 2001 Australian Census data for the area [23]. In brief, this community survey uses a sequential-cohort design with follow-up every four years for three cohorts aged 20-24, 40-44 and 60-64 years at baseline (wave one survey). The sampling frames were derived from the electoral roll in Canberra and Queanbeyan, Australia. (Note: electoral registration and voting are compulsory for Australian citizens). At baseline the 20-24 year-old cohort consisted of 1163 males and 1241 females, the 40-44 year group had 1193 males and 1337 females while the 60-64 year group comprised 1319 males and 1232 females. The cohorts respectively represented 58.6%, 64.6% and 58.3% of those contacted and in the target age ranges. The first wave of interviews for the 20-24 year cohort was in 1999-2000, 2000-2001 for the 40-44 year cohort and 2001-2002 for the oldest group. The second wave of interviews was conducted four years later for each group. In the 20-24 age cohort there were 2139 (89%) interviews, 190 refusals, seven people had died and 68 could not be contacted. In the 40-44 age group there were 2354 (93%) interviews, 135 refusals, eight deaths and 33 could not be contacted. In the 60-64 age group there were 2222 (87.1%) interviews, 234 refusals, 70 deaths and 25 could not be contacted.

### Procedure

Potential participants were selected at random from the electoral roll and were sent a letter inviting participation and describing the study. A subsequent telephone call was used to arrange a convenient location in which to conduct the interview: generally the home of the participant or the Centre for Mental Health Research. Participants used a palmtop computer to answer the survey questions, with assistance from the interviewer if required. The study was approved by the Australian National University Ethics Committee and all participants provided written consent.

### Measures

Alcohol use was assessed with the Alcohol Use Disorders Identification Test (AUDIT) [24,25]. The AUDIT was developed as part World Health Organisation project to screen for excessive drinking (hazardous or harmful patterns of consumption). The reliability (median coefficient 0.83) construct and criterion validity and excellent screening characteristics of the AUDIT have been well established, especially in English speaking groups [26]. The test-retest reliability remains under-researched but initial findings are generally supportive [26]. Weekly consumption in standard drinks was estimated from the frequency and quantity data (questions one and two) and binge drinking (question three: estimate 6.5 drinks per occasion). Based on the guidelines applicable at the start of the study, hazardous drinking was defined as 28-42 drinks per week for males and 14-28 for females. Greater levels were deemed to be harmful. Problematic use of marijuana was identified by answering "yes" to either: "In the last year have you ever used marijuana/hash more than you meant to", or "Have you ever felt you wanted or needed to cut down on your marijuana/hash use in the last year". The Physical and Mental Health Component Summary Scores of the SF-12 were used to assess physical and mental health (each has a mean of 50 (SD10) in the general population [27]), with higher scores indicating better outcomes. Compared with the original SF-36 [28], the short form (Physical Health Component and Mental Health Component scores) reproduces over 90% of the full-scale scores and has been validated against criterion groups [27].

To be eligible for inclusion in the incidence of new BI, participants had to respond "no" to the question "Have you ever had a serious brain injury where you became unconscious for more than 15 minutes?" at the wave one survey: outcomes for the 428 persons who replied "yes" and the 295 who were "uncertain" have previously been reported and are excluded from this analysis of incident cases. (Twenty eight cases with missing data on this variable were also excluded.) Incident cases at wave two were defined via two questions. BI was identified by those who replied "yes" to "Have you ever had a serious brain injury, that interfered with your memory, made you lose consciousness or caused a blood clot in your brain". Severity was defined as "moderate" for those who said that they had been admitted to hospital for head injury, with the remainder labelled as "mild". In addition, only events where the age at the time of event was within four years of the current age at the wave two survey were eligible. Of those who reported a BI at wave two, 268 were unable to recall their age at the time of the event or gave an age more than four years younger than their current age. These cases together with those reporting a BI at wave one, were excluded from both the numerator and denominator in calculating incidence rates of first time BI.

The use of medications was recorded for key areas including, depression (e.g. Aropax, Efexor Moclobemide, St John's Wort, Tryptanol), anxiety (e.g. Alprazolam, Mogadon, Valium, Vitamin B complex), pain (e.g. Codeine, Naprosyn), memory (e.g. Glutamine, Vitamin E) and sleep (e.g. Camomile, Dozile, Valium). For each area, the palmtop provided a list of commonly prescribed and over-the-counter medications. The lists ranged from five memory medications to 29 (anti) depression medications. Participants were also prompted to add any that were not already named.

### Analyses

Differences between the study groups on demographic and other characteristics were assessed with chi-square (categorical variables) or general linear models (continuous variables). Simultaneous entry logistic regression was used to model the relative risks for brain injury at wave two using wave one predictors: age, sex, alcohol category, marijuana 'problem," use of specific categories of medication (sleep, pain, anxiety, depression and memory). The development of new alcohol or marijuana problems at wave two was modelled using the same wave one variables plus marital status, smoking status, occurrence of BI and either wave one alcohol (for new marijuana problems) or marijuana problems (for new alcohol problems). To control for the five blocks of statistical inference testing, alpha was set to p < 0.01.

## Results

### Wave Two Characteristics

While there were 6715 (89.7% of wave one participants) people who completed a wave two interview, there were 6093 who were classified with some certainty as having not experienced a BI at wave one (Table 1). There were significant differences in the distribution of males and females across the cohorts and in years of education. There were also significant differences between the cohorts in the proportions using anti-depressants, anti-anxiety, pain and sleep medications, differences in history of smoking and those with marijuana use problems.

**Table 1:** Characteristics of those not reporting a head injury at wave one

		20-24 cohort N = 1916	40-44 cohort N = 2156	60-64 cohort N = 2021	*p *value
Age (years)	mean (SD)	22.6 (1.5)	42.6 (1.5)	62.5(1.5)	Not tested
Gender (female)	n (%)	1050 (54.8)	1186 (55.0)	1013 (50.1)	.002
Years of education	mean (SD)	14.3 (1.5)	14.5 (2.3)	13.9 (2.6)	<.001
Marital status					
	married/de facto n (%)	467 (24.5)	1714 (79.5)	1593 (78.9)	<.001
	separated/divorced/widowed n (%)	23 (1.2)	272 (12.6)	376 (18.6)	
	never married n (%)	1413 (74.3)	169 (7.8)	50 (2.5)	
Smoking status					
	never smoked n (%)	1124 (59.2)	1132 (52.5)	1100 (54.5)	<.001
	past smoker n (%)	216 (11.4)	651 (30.2)	734 (36.4)	
	current smoker n (%)	560 (29.5)	372 (17.3)	185 (9.2)	
Alcohol classification					
	light/niln (%)	1782 (93.9)	2014 (93.6)	1906 (94.5)	.786
	hazardous n (%)	95 (5.0)	110 (5.1)	89 (4.4)	
	harmful n (%)	21 (1.1)	27 (1.3)	21 (1.0)	
Problem marijuana use^a^	n (%)	229 (12.0)	56 (2.6)	2 (0.1)	<.001
Depression medication yes: no	n (%)	86 (4.5):	144 (6.7):	96 (4.8):	.003
	n (%)	1815 (95.5)	2011 (93.3)	1923 (95.2)	
Anxiety medication yes: no	n (%)	114 (6.0):	155 (7.2):	87 (4.3):	<.001
	n (%)	1787 (94.0)	2000 (92.8)	1932 (95.7)	
Pain medication yes: no	n (%)	1283 (67.5):	1473 (68.4):	1099 (54.4):	<.001
	n (%)	618 (32.5)	682 (31.6)	920 (45.6)	
Memory medication yes: no	n (%)	32 (1.7):	52 (2.4):	57 (2.8):	.057
	n (%)	1869 (98.3)	2103 (97.6)	1962 (97.2)	
Sleep medication yes: no	n (%)	183 (9.6):	284 (13.2):	249 (12.3):	.001
	n (%)	1718 (90.4)	1871 (86.8)	1770 (87.7)	

^a^defined via self-reported used more than planned or wanting to cut down use

### Wave Two: Incidence and Cause of First Time Brain injury

One hundred and one people reported a BI between the two surveys, of these 56 were mild (not hospitalised) and 44 were moderate (admitted to hospital) and one case had missing data and was excluded from analysis requiring a severity rating.. Table 2 shows the incidence of first-time brain injuries in the past four years stratified by sex and cohort. The overall rate (moderate and mild combined) of BI was 421.5/100000 (95% CI 338.9, 504.1) person-years (p-y) and the incidence of mild and moderate BI were respectively 230.8 (95% CI 170.4, 291.3) and 180.5 (95% CI 127.2, 233.8)/100000 p-y. In all categories, the rates were higher in the younger cohorts and in all bar one for males than females.

**Table 2:** Classification, causes and incident rates for head injuries reported at Wave two

	20-24 cohort N = 1916	40-44 cohort N = 2155	60-64 cohort N = 2021	Total N = 6092
Classification of BI				
No BI n (%)	1844 (96.2)	2137 (99.1)	2011 (99.5)	5992 (98.4)
{95% CI}	{95.3, 97.1}	{98.6, 99.5}	{99.1, 99.8}	{98.0, 98.7}
Mild BI n (%)	39 (2.0)	11 (0.5)	6 (0.3)	56 (0.9)
{95% CI}	{1.4, 2.8}	{0.3, 0.9}	{0.1, 0.6}	{0.7, 1.2}
Moderate BI n (%)	33 (1.7)	7 (0.3)	4 (0.2)	44 (0.7)
{95% CI}	{1.2, 2.4}	{0.1, 0.7}	{0.05, 0.51}	{0.5, 1.0}
Cause Mild BI				
Traffic accident n (%)	2 (5.1)	1 (9.1)	1 (16.7)	4 (7.1)
Sport n (%)	20 (51.3)	2 (18.2)	1 (16.7)	23 (41.1)
Assault n (%)	6 (15.4)	1 (9.1)	0 (0.0)	7 (12.5)
Fall n (%)	7 (17.97)	5 (45.5)	4 (66.7)	16 (28.6)
Other/don't know n (%)	4 (10.3)	2 (18.2)	0 (0.0)	6 (10.7)
Cause moderate BI				
Traffic accident n (%)	6 (18.2)	5 (71.4)	1 (25.0)	12 (27.3)
Sport n (%)	17 (51.5)	0 (0.0)	0 (0.0)	17 (38.6)
Assault n (%)	4 (12.1)	0 (0.0)	0 (0.0)	4 (9.1)
Fall n (%)	5 (15.2)	1 (14.3)	3 (75.0)	9 (20.5)
Other/don't know n (%)	1 (3.0)	1 (14.3)	0 (0.0)	2 (4.5)
Incident rate mild BI/100000 p-y				
Males	636.9	159.5	122.5	292.1
Females	401.0	108.6	24.3	177.4
Total	506.9	131.5	73.2	230.8
Incident rate moderate BI/100000 p-y				
Males	771.3	79.5	73.3	290.3
Females	140.4	86.7	24.3	84.5
Total	424.4	83.4	48.7	180.5

BI = brain injury: p-y = person years, CI = Confidence interval

Across both mild and moderate BI, sports activities was the major cause of brain injury overall (n = 40, 40%) and responsible for the majority of the injuries in the 20-24 year cohort (n = 37, 51.4%). In the oldest cohort 70% of all BI were attributed to falls while in the 40-44 year group, falls and traffic accidents were each cited in 33.3% of all BI.

Those reporting a BI showed a trend towards greater declines on the SF-12 Physical health component summary scores than those without a BI (interaction effect: *F *(1, 5889) 6.05, *p *= .014: estimated marginal means at wave one and two: BI = 50.2 (SE .7): 47.0 (SE .8), Non-BI = 51.0 (SE .1), 50.2 (SE .1)). There was no significant interaction evident for the SF-12 Mental health component summary scores (*F *(1, 5889) .657, *p *= .418).

### Risk Factors for BI by Wave Two

Compared with the 60-64 year cohort, those in the youngest group had more than seven times the risk for incurring a brain injury by wave two. In addition, males had more than twice the risk of brain injury than females. Aside from age/cohort and sex, the only other significant predictor of BI was the use of pain medication at wave one (Table 3). Neither hazardous nor harmful alcohol use nor problematic marijuana use at baseline was associated with incidence of brain injury in the following four years.

**Table 3:** Risk factors for head injury at Wave two

		Wald	df	sig	Risk	95% CI
Age groups	(60-64)					
	20-24	34.16	1	<.001	7.53	3.83-14.81
	40-44	1.79	1	.181	1.69	0.78-3.66
Gender (female)		19.9	1	<.001	2.65	1.73-4.07
Alcohol W1	("safe")					
	Hazardous	0.75	1	.387	1.40	0.64-3.14
	Harmful	0.04	1	.847	0.82	0.11-6.10
Marijuana problems W1	(no)	1.00	1	.318	1.36	0.74-2.51
Sleep medication W1	(no)	0.13	1	.716	1.13	0.60-2.12
Pain medication W1	(no)	8.11	1	.004	2.00	1.24-3.21
Anxiety medication W1	(no)	0.01	1	.900	1.06	0.43-2.63
Depression medication W1	(no)	0.030	1	.584	1.30	0.50-3.38
Memory medication W1	(no)	0.54	1	.184	2.17	0.76-6.22

W1 = Wave one data: categories in parentheses are the reference categories

### Risk Factors for Developing Substance Use Problems

There were 383 people classified via their AUDIT scores as hazardous or harmful users of alcohol at wave two, of these, 201 were new cases (i.e. they were classified as using alcohol at lower than hazardous levels at wave one on the AUDIT). There were 223 people with marijuana problems, of whom, 82 were new cases. Table 4 shows the risk factors for developing new alcohol disorders and Table 5 the risk factors for new marijuana problems. The respective analyses excluded those with alcohol or marijuana disorders at wave one, as by definition they could not develop 'new disorders' for their respective categories. Those with marijuana use problems or who were tobacco smokers at wave one, had more than twice the risk of developing alcohol problems than those who did not use these substances. Compared to the youngest cohort those aged 60-64 showed a trend towards reduced risk (approximately half) of developing new alcohol use disorders. Increased age was also strongly associated with decreased risk of developing new marijuana problems, while being male (greater than two times) and a smoker (greater than five times) increased the risk. The occurrence of a BI between W1 and W2 was not associated with increase risk of new either alcohol or marijuana problems.

**Table 4:** Risk factors for the development of new alcohol problems at wave two

			New (Wave two) Alcohol Problems			
		df	Wald	sig	Risk	95% CI
Age groups	(20-24)					
	40-44	1	1.225	.268	1.270	.832 - 1.938
	60-64	1	5.786	.016	0.513	.298 - .884
Gender	(female)	1	.546	.460	0.894	.663 - 1.204
Marital status W1	(married/de facto)					
	Separated/divorced/widowed	1	1.270	.260	.718	.404 - 1.277
	Never married	1	.277	.598	1.118	.739 - 1.691
Depression medication W1	(no)	1	.170	.680	1.150	.592 - 2.234
Anxiety medication W1	(no)	1	.018	.893	1.045	.545 - 2.004
Pain medication W1	(no)	1	.670	.413	1.142	.831 - 1.571
Memory medication W1	(no)	1	1.362	.243	0.431	.105 - 1.772
Sleep medication W1	(no)	1	.106	.745	.926	.583 - 1.471
Smoker W1	(no)	1	18.050	<.001	2.037	1.467 - 2.828
BI (W1-W2)	(no)	1	3.184	.074	2.007	.934 - 4.313
Marijuana problems W1	(no)	1	12.188	<.001	2.318	1.446 - 3.717
Alcohol problems W1	(no)	1	NiM	NiM	NiM	NiM

Items in parenthesis show the reference category:No cases used memory medication and developed marijuana problems

W1 = wave one: BI (W1-W2) = brain injury (incident case e.g. first occurrence) between Wave one and wave two: NiM = not in model

**Table 5:** Risk factors for the development of new marijuana problems at wave two

			New (Wave two) Marijuana Problems			
		df	Wald	sig	Risk	95% CI
Age groups	(20-24)					
	40-44	1	4.685	.030	0.449	.218 - .927
	60-64	1	10.475	.001	0.033	.004 - .261
Gender	(female)	1	12.967	<.001	2.507	1.520 - 4.136
Marital status W1	(married/de facto)					
	Separated/divorced/widowed	1	<.001	.996	1.003	.284 - 3.542
	Never married	1	7.387	.007	2.543	1.297 - 4.986
Depression medication W1	(no)	1	.345	.557	1.413	.446 - 4.475
Anxiety medication W1	(no)	1	.749	.387	1.573	.564 - 4.386
Pain medication W1	(no)	1	2.106	.147	.698	.430 - 1.134
Memory medication W1	(no)	1	<.001	.995	.000	.000 -^a^
Sleep medication W1	(no)	1	.081	.776	1.121	.510 - 2.467
Smoker W1	(no)	1	52.322	<.001	5.603	3.513 - 8.938
BI (W1-W2)	(no)	1	1.996	.158	2.046	.758 - 5.521
Marijuana problems W1	(no)	1	NiM	NiM	NiM	NiM
Alcohol problems W1	(no)	1	.116	.734	.859	.356 - 2.069

Items in parenthesis show the reference category: ^a ^No cases used memory medication and developed marijuana problems

W1 = wave one: BI (W1-W2) = brain injury (incident case e.g. first occurrence) between Wave one and wave two: NiM = not in model

### Sensitivity analyses

The risk factor analysis for BI by W2 was re-run including the 268 people who were unable to recall their age at the time of BI or gave an age more than four years younger than their current age. The same pattern of outcomes as shown in Table 3 was obtained except the use of pain medication was no longer significant. As with the original analysis, neither problem alcohol nor marijuana use was significant. Sensitivity analyses of the risk factors for the development of a) alcohol and b) marijuana problems were also conducted. For the development of alcohol problems the results were similar to those shown in table 4. For marijuana problems the results were similar to the original analysis except those with BI were at increased risk of developing a marijuana problem by W2 (2.0 95% CI 1.07, 3.73).

## Discussion

Data from this longitudinal community follow-up showed marked differences in the incidence of self-reported brain injuries across age groups and gender: in the youngest males the rate was over 771 per 100000 person-years whilst in the oldest females it was under 25 per 100000 p-y. The major causes of injury also varied by age group and with severity of brain injury. Clinical studies have reported links between brain injuries and alcohol/substance use disorders [13-15] but in this community sample no clear relationship was found between alcohol or marijuana problems and the occurrence of brain injury or for the development of these problems post brain injury.

Nationally representative population survey data from the USA reported the incidence of mild and moderate BI to be 618 per 100000 p-y [11]. However, that study sampled across a wide age range and included respondents younger than 24 years. This young age range has previously been found to have the greatest rate of BI requiring hospital treatment (e.g. <5 years 1115, 5-14 years 733.3 per 100000 p-y) with the incidence rate falling across the age range such that those aged 65-74 years have 217.9 hospitalised brain injuries per 100000 p-y [20]. The current data reveal a similar pattern with rates falling with increasing age, albeit with a rate generally lower than that cited for the USA.

The literature shows that among those experiencing TBI, the cause of injury is predominately vehicular trauma and fall, particularly in this adult age range [17,18,29]. By comparison, American data show that in BI categorised as mild or moderate there is an increase in the prevalence of assaults (especially young adults), sports related and other accidental injuries [10,20]. Among Australian general hospital-treated brain injuries, 25% are sports related [30] while among those with severe TBI this falls to less than 5% [17]. In the current study, sports-related injuries was the largest category for those with mild brain injuries but among those with moderate injuries the proportion of traffic related injures increase markedly.

An important finding from our study was that neither baseline problematic use of alcohol nor marijuana was associated with incurring BI in the subsequent four years. In contrast, previous research suggests that in a clinical group, a greater proportion (about three times) of those who incur brain injury will be heavy drinkers than would be expected in the sample was drawn from the USA general population [31]. In addition, previous research also suggests that a greater proportion of those experiencing TBI use illicit drugs [14,31]. The relationship between recent alcohol use (i.e. intoxication at the time of event) and TBI is more difficult to quantify as the probability of having blood alcohol content assessed after injury appears to vary by severity and external cause of injury [32]. However, acute intoxication appears to be associated with a poorer prognosis on some measures of memory and visuospatial functioning following brain injury [33]. Epidemiological studies have not reported on the relationship between alcohol/substance use disorders and brain injuries [10,11,20]. Therefore, we speculate that the reason for the difference between our findings and those of previous studies with respect to alcohol and drug use is that these samples were drawn from hospitals/rehabilitation programs. The link between alcohol use and motor vehicle accidents is well documented [34,35] and motor vehicle accidents are a common cause of severe brain injury. In the community sample, most of the injuries were of a less severe nature where alcohol or other drug use may exert a smaller influence.

It has previously been noted that, beyond the age of approximately 65 years, increasing age is associated with increased incidence of BI - thus those aged 85+ have a 10 fold rate of BI requiring hospital treatment than those aged 60-64 years and nearly six times the rate of intracranial injury [10,36]. Therefore, we expect that when the 60-64 year cohort is next interviewed (when they will be aged 68-72 years), an increased rate of injury will be found. Jamieson and Roberts-Thomson reported an incidence of 268.9 and 321.5 brain injuries per 100000 in those aged 60-64 and those aged 65-69 years respectively: markedly higher than the figures cited in the current study. However, these covered all types of BI including superficial injury whereas our key question asked for serious injury and specified that this required interference with memory, loss of consciousness and/or blood clot to the brain.

Those who incurred a BI reported a trend towards worse physical health outcomes than those without a BI. However, it should be noted that the magnitude of this difference was small (three points or approximately 1/3^rd ^of a standard deviation on the SF-12 Physical health component summary score). In comparison, group norms for those with a serious *versus *minor physical condition show a difference of over eight points on the SF-12 Physical health component [27]. The difference observed in this study was of a similar magnitude to that reported from a UK sample across different age groups (e.g. mean scores for those aged 18-44, 45-64 and 65-74 years were 53.4, 49.1 and 45.3 respectively [37]). It has been suggested that a change of five points on the SF-36 total score is the minimum to be of clinical importance [38], although there appear to be large differences in minimum change scores required to be clinically important for different disorders [39]. Thus, there is likely to be limited clinical significance in the physical heath differences of those with and without BI, particularly mild BI, in our sample.

Use of pain medication at wave one was a risk factor for BI. We speculate that this is a marker for those who undertake more risky activities and acquire minor injuries that require pain medication. Other medications such as sleeping pills, anti-depressants and anxiolytics that may cause drowsiness, impaired concentration or interfere with the operation of machinery/driving were not implicated in BI.

### Limitations

Across the two waves of the study there have been changes to the questions on brain injury. In wave one, the questions opened with serious head injuries defined as being unconscious for more than 15 minutes. At wave two, participants were asked about head injuries that required treatment in an emergency department, hospital or by a general practitioner before they were asked about serious head injuries. As a result some people, who did not report a head injury at wave one, did report a head injury at wave two but reported an age of occurrence indicating that it was prior to wave one. We excluded events where an age could not be recalled: this conservative approach would be likely to lead to an underestimation of the true incident rates. The first sensitivity analysis showed that inclusion of this sub-group did not have a major impact on the identification of risk factors for BI. Further, their inclusion did not alter the factors for developing alcohol disorders. However, the third sensitivity analysis showed that when this sub-group was included, those with BI were at increased risk of developing a marijuana use problem. A possible explanation is that marijuana use has been shown to impair memory, especially on 'real world' memory tasks which may lead to less accurate recall of when the BI occurred [40,41]. A further potential influence of the change in wording is that there may be some people who at wave one had an injury that resulted in a LOC of less than 15 minutes but who were admitted to hospital for their injury, and should not be in the denominator for incident cases.

The main limitation of a community survey on this topic is the lack of data on the severity of the injury, in particular descriptive measures such as Glasgow Coma Scores [42] and standard outcome measures like the Glasgow Outcome Scale [43,44]. However, given the mild nature of most of the reported injuries, with over 50% not attending hospital for an injury that caused loss of consciousness, blood clot or impaired memory, assessment may require more detailed and subtle measures. Finally, the low counts in some cells, especially for the oldest cohort means that the estimated incidence rates for this group maybe unstable.

## Conclusions

In Australia there were an estimated 21 800 TBI cases in 2004-05 that were treated in hospital [45]. Health outcomes for Australian patients hospitalised with moderate to severe BI have been documented [17] but the incidence and subsequent outcomes for community reported brain injuries remains under-researched. Our findings suggest that BI associated with sport constitute a significant proportion of BI occurring in the community. It is possible that individuals who experience BI due to sport have different help-seeking behaviour to those experiencing BI due to substance use or traffic accidents. A better understanding of the different sub-groups in the population at risk of BI is required to enable the design and implementation of prevention programs.

## Competing interests

The authors declare that they have no competing interests.

## Authors' contributions

KA and PB developed the original head injury paper. RT developed the follow-up paper. Analysis and initial manuscript production were by RT. Extensive comment and editorial work by KA and PB. All authors read and approved the final manuscript.

## Pre-publication history

The pre-publication history for this paper can be accessed here:

http://www.biomedcentral.com/1471-2458/10/171/prepub
